# Clinical Significance of Plasma Levels of Gluconeogenic Amino Acids in Esophageal Cancer Patients

**DOI:** 10.31557/APJCP.2020.21.8.2463

**Published:** 2020-08

**Authors:** Mahsa Taherizadeh, Masoud Khoshnia, Sedigheh Shams, Zahra Hesari, Hamidreza Joshaghani

**Affiliations:** 1 *Metabolic Disorders Research Center, Golestan University of Medical Sciences, Gorgan, Iran. *; 2 *Golestan Research Center of Gastroenterology & Hepatology, Golestan University of Medical Sciences, Golestan, Gorgan, Iran. *; 3 *Children Medical center, Tehran University of Medical Sciences, Tehran, Iran. *; 4 *Laboratory Sciences Research Center, Golestan University of Medical Sciences, Gorgan, Iran. *; 5 *Department of Laboratory Sciences, Faculty of Paramedicine, Golestan University of Medical Sciences, Gorgan, Iran. *

**Keywords:** Esophageal cancer, amino acid, gluconeogenic pathway, HPLC

## Abstract

**Objective:**

Metabolic processes in the body of people with and without esophageal cancer (EC) are significantly different. Therefore, changes in the metabolism of amino acids in the body of EC patients can lead to metabolic disorders, such as increased gluconeogenesis. The aim of this study was the comparison of the plasma levels of gluconeogenic amino acids between patients with EC and the control group.

**Methods:**

Plasma samples of 37 patients with EC who were selected before any treatment or surgery, and 37 healthy adults who did not have history of family cancer and malignant diseases were taken. Analysis of the plasma levels of amino acids including, alanine, asparagine, aspartate, glutamate, glutamine, glycine, serine, arginine, histidine, methionine, threonine, valine, tyrosine, isoleucine, phenylalanine, tryptophan was done by High Performance Liquid Chromatography (HPLC) based on reverse-phase-chromatography. Data analysis was done by SPSS-16 software.

**Results:**

In the patient group the mean age ± SD was 63±13.64 and 21 (56.8%) were male.The plasma levels of the alanine, asparagine, histidine, methionine, threonine, valine amino acids in the patients with esophageal cancer was significantly reduced and glycine was increased (p-value<0.05).

**Conclusion:**

Gluconeogenic amino acids are the main precursor of glucose synthesis in the gluconeogenesis pathway. Cancer cells need more energy to grow and multiply, and glucose is used as the main fuel for cells. Given the importance of metabolic pathways in cancer cells, more detailed studies at the molecular level can provide new insights into early detection and appropriate treatment strategies for cancer.

## Introduction

Cancer is one of the leading causes of death in the world (Niya et al., 2017) and Esophageal cancer (EC) is a serious and deadly disease and is the eighth most prevalent cancer and the sixth leading cause of death from cancer worldwide (Ma et al., 2014). Because of the lack of symptoms in the early stages of the disease, most patients in the advanced stage notice the disease, which is accompanied by diffused metastasis. Despite recent therapeutic advances, the five-year survival rate of these patients is less than 20% (Lambert and Hainaut, 2007) and only 5% of these patients are diagnosed (Huang and Yu, 2018). The prevalence of EC varies in different parts of the world and it reaches the highest rate of 800 cases per 100,000 people in some regions, and in the world the widest rate of EC is in such areas as southern Russia, northern China, and northern Iran (Lin and Chang, 2010). According to the studies, Golestan province in Iran has one of the highest mortality rates due to this disease in the world. In addition, studies have shown that EC is more prevalent in men, and there are different theories about the high prevalence of this cancer in Golestan province (Roshandel et al., 2012). Race, diet, drinking hot tea, and smoking are some of the factors contributing to the spread of the disease in this region (Kamangar et al., 2007). Esophageal cancer includes two types of Esophageal Squamous Cell Carcinoma (ESCC) and Esophageal Adenocarcinoma (EAC). Although ESCC accounts for 95% of cases of esophageal cancer worldwide, EAC is currently on the rise in the Western world with a high prevalence (Lin and Chang, 2010). 

Recent studies have examined the diverse roles of amino acids in tumors (Lieu et al., 2020). Amino acids are substantial organic compounds, and play a major role as building blocks in proteins and as intermediaries in metabolism (Altman and Dang, 2012). They also play a role in energy production, the synthesis of nucleosides, and the maintenance of redox balance in cancer cells and amino acid derivatives are also involved in the regulation of epigenetics and immune responses associated with tumor formation and metastasis (Vettore et al., 2019). Gluconeogenic amino acids are amino acids that produce pyruvate or one of the metabolites of the Krebs cycle (α-ketoglutarate, succinyl-CoA, fumarate and oxaloacetate), which are capable of conversion to glucose (Grasmann et al., 2019). These amino acids include arginine, glutamine, histidine, proline, glutamate, isoleucine, methionine, threonine, valine, phenylalanine, tyrosine, asparagine, aspartate, alanine, cysteine, glycine, serine, and tryptophan (Young and Ajami, 2001; Brosnan, 2003). The concentration of some serum amino acids has a special relationship with the type of tumor and its effect on the host’s metabolism (HOLT Jr et al., 1963). Extensive studies have been published that show the reduced concentration of gluconeogenic amino acids in cancer because the liver accelerates gluconeogenesis to supply its required glucose in cancers (Wahren et al., 1972; Russell et al., 1981). However, such factors as carbohydrate intolerance also lead to changes in the concentration of amino acids (Wahren et al., 1972). Studies also show that metabolic pathways in cancer cells are used for multiplication and regrowth of tumors, which in turn increases fuel consumption in cells(Niya et al., 2018a; Grasmann et al., 2019). In the 1920s, the Nobel Prize winner, Warburg, explained that tumor masses, even in non-hypoxic conditions, inadvertently consume glucose and produce lactate, increasing glucose uptake into cancer cells (Warburg, 1926). Glucose is the most important source of fast access to energy.Glucose is broken down into pyrethrins by cytosolic glycolysis and transported into the mitochondria, then oxidized completely in the carbon dioxide cycle and generates chemical energy. Reversible reduction of pyruvate to lactate allows anaerobic glycolysis. Gluconeogenesis is the process of glucose synthesis by non-carbohydrate precursors, one of which is gluconeogenic amino acids. Gluconeogenesis occurs primarily in the liver, kidneys, intestines, and skeletal muscles (Hui et al., 2017). In gluconeogenesis, pyruvate is carboxylated to oxaloacetate by the pyruvate carboxylase enzyme, which is itself transported through the malate into the cytosol and is consumed to make glucose 6-phosphate, and it is eventually converted to glucose. Enzymes of this pathway include Phosphoenolpyruvate carboxykinase, and Fructose-1,6-bisphosphate (Aldolase A) (Berg et al., 2002; Wang and Dong, 2019). Nowadays, amino acids are one of the most suitable candidates for concentrated metabolomic studies and play important physiological roles in the body (Miyagi et al., 2011) and recent studies have also shown that restrictions on some amino acids play a role in cancer interventions (Kang, 2020). Due to the high prevalence of EC in north of Iran, the current study was conducted aiming at evaluating and measuring the plasma concentration of gluconeogenic amino acids in patients with esophageal cancer in this area. 

## Materials and Methods

*Study population*

Samples were taken from 37 patients with Esophageal Cancer referred to Research Center of Gastroenterology & Hepatology of Golestan and they were selected before any treatment (chemotherapy, radiation) and surgery. Patients with other metastatic and metabolic diseases were excluded. Also, this study was confirmed by Ethics Committee of Golestan University of Medical Sciences. The control group included people with perfect health, and those who did not have history of family cancer and malignant diseases. 5 ml of the fasting patient’s peripheral blood vessels were taken in tubes containing Ethylenediaminetetraacetic acid (EDTA) and then samples were separated by 1,000g centrifugation for 10 minutes and was frozen at -80°C until analysis.

Analysis of gluconeogenic amino Acids (alanine, asparagine, aspartate, glutamate, glutamine, glycine, serine, arginine, histidine, methionine, threonine, valine, tyrosine, isoleucine, phenylalanine, tryptophan) was done by High Performance Liquid Chromatography (HPLC) device model KNAUER (Germany). This study was conducted based on reverse-phase chromatography (RP-HPLC) and gradient method with flow rate as 1 and ph = 7.02 (CJ, 1982). In this step, 200 microliter sample was mixed with 50 microliter standard homoserine and 800 microliter methanol and incubated for 5 min at 4°C and then the supernatant was centrifuged for 5 minutes at 4,000 rpm and 250 microliter of it was mixed with 100 microliter borate buffer (keeping ph) was vortex for 5 seconds and 25 microliter normal hydrochloric acid (HCL) 75% was added to it and again 5 seconds vortex was done. Then 50 microliter of the resulting solution was added with 200 microliter of solution A and 5 seconds vortex was done. After that 20 microliter of solution was taken by syringe Hamilton and injected to HPLC device. Plasma levels of alanine (ALA), asparagine (ASN), aspartate (ASP), glutamate (GLU), glutamine (GLN), glycine (GLY), serine (SER), arginine(ARG), histidine (HIS), methionine (MET), threonine (THR), valine (VAL), tyrosine (TYR), isoleucine (ILE), phenylalanine (PHE), tryptophan (TRP) amino acids were measured by HPLC for 60 minutes.

*Statistical analysis*

Data were statistically analyzed using SPSS-16 software and standard deviation and mean concentration of gluconeogenic amino acids in the patients with EC and the control group was measured. P-values smaller than 0.05 was considered statistically significant. To test the normality of the data, the test Kolmogorov-Smirnov was used. Analysis of the amino acids with normal distribution was dane by t-test (SER, GLN, GLY, ALA, TRP, MET, VAL, ILE). On the other hand, amino acids that had no a normal distribution the Mann-Whitney test was selected for measuring significant difference in plasma level of gluconeogenic amino acids in patients with EC and healthy people.

## Results

The mean age ± SD in the patient group was 63±13.64 and 21 (56.8%) were male; Of 37 control group, the mean age ± SD was 64.24±13.08 and %54.1 were male. Plasma levels of the alanine, asparagine, aspartate, glutamate, glutamine, glycine, serine, arginine, histidine, methionine, threonine, valine, tyrosine, isoleucine, phenylalanine, tryptophan amino acids in patients with esophageal cancer was measured. Finally, the results showed that the plasma levels of alanine, asparagine, histidine, methionine, threonine, valine, Isoleucine in patients with esophageal cancer compared to the healthy group was significantly reduced and glycine level was significantly increased (p-value <0.05) (Tables 1 and 2).

**Figure 1:**
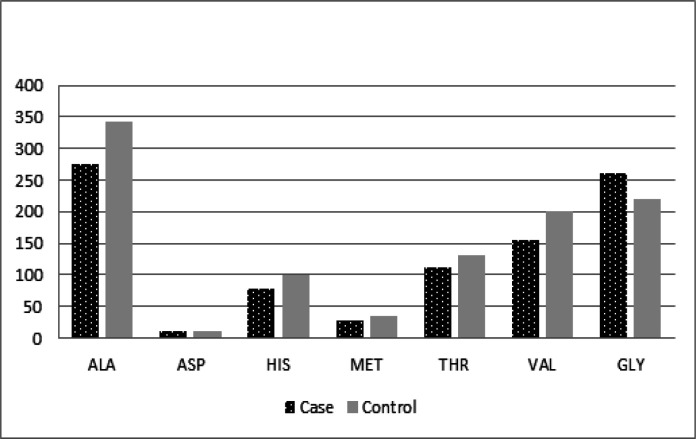
Changes of Alanine (ALA), Aspartate (ASP), Histidine (HIS), Methionine (MET), Threonine (THR), Valine (VAL), Glycine (GLY) Levels in the Patient Group and the Control Group

**Table 1:** The Average Concentration of Gluconeogenic Amino Acids in the Case and Control Groups with t- test

Amino Acid (µm)				Glutamine	Serine	Glycine	Alanine	Tryptophan	Methionine	Valine	Isoleucine
Age	Case (N=37)	Pearson correlation		-0.056	-0.078	-0.307	-0.013	0.481	0.211	-0.229	-0.08
		P-value		0.74	0.644	0.064	0.939	0.003	0.21	0.174	0.638
	Control (N=37)	Pearson correlation		0.137	-0.411	-0.066	0.034	0.148	-0.073	-0.013	-0.089
		P-value		0.42	*0.012	0.699	0.842	0.382	0.666	0.939	0.602
Sex	Case (N=37)	Mean ± SD	male	477.74±98.19	120.59±47.31	235.64±84.36	271.88±81.96	15.58±2.85	29.75±8.38	158.05±36.53	47.27±12.72
			fmale	470.71±101.09	124.46±37.33	293.41±84.08	280.79±99.31	15.58±2.02	25.53±6.25	151.79±40.07	45.03±14.04
	Control (N=37)	Mean ± SD	male	503.62±85.13	124.68±53.92	218.88±69.11	362.88±96.82	16.56±2.95	35.12±12.29	210.42±56.58	67.73±24.87
			fmale	532.82±112.79	121.34±42.12	22.03±45.01	318.17±107.14	16.08±1.98	32.21±8.89	191.27±56.86	52.48±17.65
A comparison of amino acids with test-t		Case	Mean ± SD	474.7±98.12	122.26±72.76	260.62±89.19	275.73±88.66	15.58±2.49	27.93±7.73	155.35±37.68	46.3±13.16
		(N=37)									
		Control (N=37)	Mean ± SD	517.04±98.47	123.15±48.22	220.33±58.52	342.34±102.76	16.34±2.53	33.78±10.82	201.62±56.74	59.1±22.43
		p-value		0.689	0.61	*0.031	0.271	0.902	*0.019	*0.006	*0.007
		Equal variances assumed		0.068	0.934	0.025	0.004	0.198	0.009	0.0001	0.004
		Equal variances not assumed		0.068	0.934	0.025	0.004	0.198	0.009	0.0001	0.004

**Table 2:** Average Concentration of Gluconeogenic Amino Acids in the Case and Control Groups with Mann-Whitney Test

Amino Acid(µm)				Aspartate	Glutamate	Asparagine	Histidine	Threonine	Arginine	Tyrosine	Phenylalanine
Age	Case (N=37)	Pearson correlation		-0.032	-0.039	-0.05	0.063	0.063	-0.076	0.286	0.075
		P-value		0.851	0.821	0.769	0.713	0.713	0.657	0.086	0.66
	Control (N=37)	Pearson correlation		-0.352	-0.295	-0.012	-0.007	-0.258	-0.065	0.1	-0.029
		P-value		*0.033	0.076	0.944	0.967	0.123	0.704	0.554	0.866
Sex	Case (N=37)	Mean ± SD	Male	10.55±11.38	107.54±57.23	33.66±8.2	73.68±26.87	101.32±46.56	40.53±16.95	59.56±19.09	55.93±8.41
			Fmale	10.1±5.87	137.86±86.61	41.96±16.73	80.68±28.99	124.91±63.65	48.74±35.15	52.91±14.11	63.11±20.53
	Control (N=37)	Mean ± SD	Male	8.93±5.81	102.1±61.29	43.3±14.28	96.1±17.46	128.99±50.18	60.27±42.05	64.48±20.34	59.76±19.71
			Fmale	10.71±6.74	74.5±40.73	39.18±8.97	106.04±29.82	135.28±50.08	44.17±25.1	63.45±28.67	64.07±21.19
	P-value		Male	0.969	0.514	*0.006	*0.001	0.064	0.054	0.498	0.584
			Fmale	0.843	0.021	0.871	0.004	0.305	0.928	0.16	1
A comparison of amino acids with Mann-Whitney test.	Case (N=37)	Mean ± SD		10.35±9.29	120.65±71.95	37.25±13.09	76.71±27.63	111.52±55.07	44.08±26.29	56.69±17.22	59.04±15.09
	Control (N=37)	Mean ± SD		9.75±6.23	89.42±53.98	41.41±12.16	100.67±24.11	131.88±49.54	52.87±35.77	64.01±24.17	61.74±20.23
	P-value			0.987	0.054	*0.044	*0.0001	*0.047	0.122	0.155	0.527

## Discussion

Upper gastrointestinal cancers are still one of the leading causes of cancer deaths in northern Iran (Niya et al., 2018b). According to the National Cancer Registry Report 2007 in Iran, esophageal cancer with 6.4% was the most prevalent type of cancer in the 7th century in Iran(Aledavood et al., 2011). Metabolism of amino acids and proteins in the body of cancer patients causes metabolic disorders, such as increased gluconeogenesis and increased protein synthesis in the liver (Lundholm et al., 1976).

 In this study, we looked at in the plasma concentration changes of gluconeogenic amino acids in esophageal cancer patients. The results showed a significant reduction in a large number of gluconeogenic amino acids, such as asparagine, alanine, threonine, methionine, valine, and histone in esophageal cancer patients, indicating an increase in gluconeogenesis in cancer patients. In addition, these amino acids are metabolic mediators of some important biochemical reactions in the body. Clarke et al., 1978). Their studies showed that the pattern of changes in amino acids varies in cancer patients and that some gluconeogenic amino acids in patients with the disease compared to the control group was decreased significantly .Cascino et al., (1988). also measured the plasma levels of gluconeogenic amino acids in 24 healthy individuals and 42 untreated cancer patients. Patients were divided into different groups according to tumor spread, glucose tolerance, and malnutrition and the results of their study showed plasma levels of gluconeogenic amino acids in these patients were significantly decreased under the influence of malnutrition, while their levels were independent of tumor spread and glucose intolerance, which may be due to increased muscle protein turnover in malignancies.

 In addition, Glass et al., (1986). assessed plasma levels of amino acid alanine (ALA) in patients with cancer, and Hong et al., (2014). studied plasma levels of ALA in EC patients. Totally, the findings of both studies showed a significant reduction of plasma levels of amino acid ALA. In our study, ALA was also significantly reduced in EC patients compared to healthy individuals. These observations may indicate that a malignant tumor is associated with an increase in the glycolytic cycle, which is also associated with an increased need for protein synthesis in the tumor, leading to an increase in alanine consumption by tumor tissue. Subsequently, plasma levels of alanine are decreased in people with esophageal cancer. Moreover, pyruvate conversion is catalyzed by the alanine aminotransferase (ALT) enzyme, so further studies are needed to confirm ALT activity and find basic biochemical pathways for amino acid alanine in esophageal cancer. Miyagi et al., (2011) in a study of people with lung cancer using HPLC, and Hong et al., (2014) in their study on esophageal cancer patients, found a significant decrease in plasma levels of the amino acid histidine (HIS). Our study also showed a significant reduction in HIS, which, in addition to consuming histidine in the gluconeogenesis pathway, may be due to the fact that HIS, as a source of carbon, allows the conversion of formanimo to tetrahydrofolate, which contributes to the synthesis of Purines and Pyrimidines. Since cancer cells are active nucleic acids metabolically, HIS is over-absorbed into cancerous tissue and its consumption is significantly increased, leading to lower plasma histidine levels, which could be another reason for the decrease in plasma HIS concentrations in cancer patients. Valine (VAL), in addition to the contribution to gluconeogenesis, is also consumed as a substrate for the synthesis of proteins and nucleic acids and other substances, and this leads to higher consumption of this amino acid in cancer patients. Also, Yousuke et al., (2010) measured the plasma levels of some amino acids, such as histidine and threonine, in colorectal cancer patients using HPLC. They found the significant reduction in the plasma levels of these amino acids. In 2018, Suzuki et al., (2018) studied patients with gynecological cancer with an abnormal profile of amino acids and examined the profile of amino acids in patients before and after treatment and their results showed that profiles of amino acids in women’s cancers have a specific pattern, even in patients who do not have signs of cancer, and these changes can be used to diagnose the disease early. On the other hand, Synakiewicz and et all examined the role of plasma levels of amino acids in the development of childhood cancer. In this study, 77 children with cancer, including 47 patients with hematological disease and 30 patients with solid tumors, were examined and their results showed plasma concentrations of some Amino acids in these children have a specific pattern of changes that can be used as an innovative approach to early detection of childhood cancer (Synakiewicz et al., 2017). Also, Junjun et all used changes in plasma concentrations of amino acids and acyclacarnitine to screen for lung cancer, and their results showed that the plasma concentrations of a number of amino acids in these patients significantly changed. Therefore, their results demonstrated that it could be used to diagnosis of lung cancer (Ni et al., 2019).

Recently, various studies have been carried out on changes in the metabolic profile of tumor tissue compared to healthy tissue, which significant changes were observed in energy metabolism between tumor tissue and healthy tissue due to increased oxygen supply to tumor tissue.

In conclusion, studies in the field of gluconeogenesis in cancers show that cancer cells can lead to the growth and survival of specific metabolic reactions by activating or regulating the activity of gluconeogenesis pathway enzymes that normally have specific functions in the cell. The enzymes involved in gluconeogenesis, which mediate the biosynthetic pathways of important anabolites and regulate various cellular functions, have different functions in the cell. So they may provide significant therapeutic goals in the treatment of cancers.
